# The relationship between circadian blood pressure patterns and electrical risk score in patients with hypertension

**DOI:** 10.1097/MD.0000000000044511

**Published:** 2025-09-12

**Authors:** Ali Nizami Elmas, Halil Fedai, Zulkif Tanriverdi

**Affiliations:** a Clinic of Cardiology, Menderes State Hospital, Izmir, Turkey; b Department of Cardiology, Faculty of Medicine, Harran University, Sanliurfa, Turkey.

**Keywords:** ambulatory blood pressure monitoring, electrical risk score, electrocardiography, non-dipper hypertension

## Abstract

Non-dipper hypertension is defined as a decline in nighttime blood pressure (BP) of <10% compared to daytime measurements. This condition is associated with poor prognosis. The electrical risk score (ERS) is a novel risk scoring and can easily be calculated from the surface electrocardiography. Although many Electrocardiography (ECG) parameters have been investigated separately in patients with non-dipper hypertension, no study has evaluated ERS in these patients to date. Our study aimed to assess the relationship between non-dipper hypertension and ERS. A total of 302 patients diagnosed with hypertension were enrolled in this retrospective study. The ERS parameters included: heart rate > 75 bpm, presence of left ventricular hypertrophy, QRS transition zone ≥ V4, frontal QRS-T angle > 90°, corrected QT Interval (QTc) interval > 450 ms in males and > 460 ms in females, and Tp-e interval > 89 ms. The number of abnormal ECG signs was used to calculate the ERS. Baseline characteristics were comparable between the non-dipper and dipper hypertension groups. However, patients with non-dipper hypertension had significantly higher ERS compared to patients with dipper hypertension (1 [1–2] vs 2 [1–3], *P* < .001). In addition, ERS was positively correlated with nighttime systolic blood pressure (*r* = 0.210, *P* < .001). Multivariate logistic regression analysis demonstrated that ERS was the independent predictor of non-dipper pattern (OR: 1.935, 95% CI: 1.543 to 2.426, *P* < .001). ERS is a novel and easily obtainable electrocardiographic risk scoring system. Our study shows that ERS may be used as a beneficial and effective tool for identifying the non-dipper pattern in patients with hypertension.

## 1. Introduction

Persistent high blood pressure (BP) in systemic arteries is a distinctive feature of hypertension, and hypertension is the most significant modifiable risk factor for morbidity and mortality worldwide.^[1]^ However, the presence of difficulties in the management and treatment of hypertension causes an inability to control and monitor the condition.^[1]^ Ambulatory BP monitoring (ABPM) has an important role both in the diagnosis and monitoring of hypertension and cardiovascular risk assessment. Dipper pattern is defined as a diurnal variation in BP, with a fall of more than 10% during sleep compared to daytime measurements.^[2]^ On the other hand, patients exhibiting a reduction in sleep BP of <10% compared to daytime values are defined as non-dipper hypertension.^[1]^ Numerous studies have demonstrated that non-dipper hypertension is associated with an increased risk of cardiovascular morbidity and mortality.^[1–4]^ Although the relationship among non-dipper hypertension, increased end-organ damage, and higher cardiovascular event rates remains unclear, it is thought to be associated with increased inflammation and high platelet activity.^[5]^

Various ECG variables, such as cardiac depolarization/repolarization markers and resting heart rate, have been found to be associated with increased risk of ABPM abnormalities.^[6–10]^ However, previous studies focusing on individual ECG parameters, such as QRS duration, QT interval, or voltage criteria, have generally shown limited discriminatory ability and inconsistent findings across different populations. Conversely, the ERS integrates depolarization, repolarization, and left ventricular hypertrophy signals, thus providing a more comprehensive and potentially clinically applicable measure of cardiovascular risk. However, the discriminatory power of individual ECG parameters is limited, leading to the exploration of new approaches that utilize ECG as a risk predictor.^[11]^ Recently, various scoring systems have been defined to assess mortality and morbidity using ECG. One of these scores is the electrical risk score (ERS), which consists of 6 parameters derived from the ECG as follows: I) heart rate, (II) electrocardiographic presence of the left ventricular hypertrophy, (III) QRS transition zone, (IV) frontal QRS-T angle, (V) corrected QT interval (QTc), and (VI) T peak to T end interval (Tp-e).^[12,13]^ Scoring is based on the presence of these parameters, with abnormal and normal electrical values assigned scores of 1 and 0, respectively.^[12,13]^ Patients can receive a minimum score of 0 and a maximum score of 6.

Although many ECG parameters have been investigated separately in patients with non-dipper hypertension, no study has evaluated ERS in these patients to date. In the current study, it was aimed to investigate the clinical utility of ERS for predicting non-dipper status in patients with hypertension.

## 2. Materials and methods

### 2.1. Participants

This retrospective single-center cohort study included 302 hypertensive patients who underwent 24-hour ABPM between September 1, 2023, and September 30, 2024. The definition of hypertension was based on the current guidelines.^[1]^ Measurements taken with 24-hour ABPM defined dipper hypertension as a decrease of 10% or more in nighttime BP compared to daytime values, and non-dipper hypertension as a decrease of <10%. The study included patients diagnosed with hypertension according to the ESC 2024 hypertension guidelines,^[1,14]^ aged 18 and older, with baseline ECG and demographic data available. The exclusion criteria were defined as II/III-degree atrioventricular block, complete and incomplete left bundle branch block, atrial fibrillation, and patients without an ECG. A permission was obtained from the local ethics committee (Date: October 23, 2024; Approval No.: 2024/184).

### 2.2. Electrocardiographic analysis

All patients underwent a standard 12-lead ECG during outpatient visits in the supine position with a paper speed of 25 mm/s and a calibration of 10 mm/mV using standard limb and chest leads. A filter range of 0.16 to 100 Hz, a speed of 25 mm/s, and a height of 10 mm/mV were used for the ECG recording. A team of 2 cardiologists, working independently, conducted an analysis of all ECGs, utilizing the measurements that were provided by the ECG device. The ERS parameters were examined in ECGs. ERS parameters such as heart rate, QTc interval, Tp-e interval, electrocardiographic presence of left ventricular hypertrophy, QRS transition zone and frontal QRS-T angle were evaluated in detail and ERS was calculated.^[13,15]^

The heart rate was obtained by dividing 1500 by the number of small squares between 2 R-Rs. The heart rate > 75 was calculated as 1 point, and < 75 heart rate was calculated as 0 point. The QT interval was measured as the time from the onset of the QRS wave to the termination of the T wave, and Bazett formula was used to compute the QTc interval (QT Bazett: QT/ √RR).^[14,16]^ QTc interval was calculated as 1 point if it was > 450 ms in men and > 460 ms in women, and 0 points if it was lower than these values. The Tp-e interval was defined as the interval from the peak of the T wave to the end of the T wave on the ECG.^[17]^ Tp-e interval > 89 ms was calculated as 1 point, and < 89 ms as 0 point. Sokolow-Lyon voltage criteria was calculated as the sum of the highest R wave in V5/V6 and the deepest S wave in V1/V2. In addition, R wave amplitude > 25 mm in V5 or V6, the sum of the R wave in D1 and the S wave in D3 > 25 mm, and the R wave in aVL > 13 mm or the R wave in aVF > 20 mm used for the criteria.^[16]^ If one of these criteria was met, it was calculated as 1 point, and if it was not met, it was calculated as 0 points. The precordial lead in which the R wave is greater than or equal to the S wave has been defined as the QRS transition zone.^[18,19]^ A QRS transition zone of ≥ V4 was calculated as 1 point, and < V4 was calculated as 0 points. Frontal QRS-T angle was defined as the absolute value of the difference between ventricular depolarization (QRS axis) and repolarization (T axis). The frontal QRS-T angle was measured by taking the absolute value of the difference between the QRS axis and the T axis. If the difference was >180°, these degrees were subtracted from 360° and the frontal QRS-T angle was recalculated.^[20]^ While measuring this angle, the calculation was made using the QRS and T axes written in the automatic report section of the ECG device. A frontal QRS-T angle > 90° was calculated as 1 point, and < 90° was calculated as 0 point.^[13]^ Scoring was done severally for each parameter, and the total score was defined as ERS (minimum score: 0, maximum score: 6).^[13]^

### 2.3. Statistical analysis

SPSS 21.0 was used for statistical analyses. The Kolmogorov–Smirnov test was applied to determine the normality of data. Continuous variables were expressed as mean ± SD or median (25th–75th interquartile range). The comparison of 2 independent variables was made by *t*-test or Mann–Whitney *U* test according to normality. Spearman correlation coefficients were used to determine the correlation between ERS and nighttime systolic blood pressure (SBP). Logistic regression analysis was performed using univariate and multivariate models. Variables that have an unadjusted *P* value of < 0.1 in the univariate analysis were selected as the possible risk factors and included into the full multivariate model, and independent predictors were determined according to the multivariate logistic regression analysis. The optimal cutoff value and area under curve (AUC) of the ERS was determined with receiver operating characteristic (ROC) (Youden index) curve analysis. A *P* value < .05 was defined as statistically significant.

## 3. Results

### 3.1. Clinical and demographic characteristics and laboratory findings

A total of 302 patients were included in this study. According to the 24h Holter recording, 181 (59.9%) patients had dipper hypertension while 121 (40.1%) patients had non-dipper hypertension. Baseline characteristics were similar between the dipper and non-dipper hypertension groups. However, patients with non-dipper hypertension had significantly higher night SBP (132.6 ± 6.4 vs 122.5 ± 5.5, *P* < .001), night diastolic blood pressure (DBP) (78.8 ± 8.7 vs 75.1 ± 6.5, *P* < .001) and left ventricular mass index (LVMI) (80.6 ± 24.5 vs 74.7 ± 23.8, *P* = .039) (Table 1).

**Table 1:** Comparison of baseline characteristics of the study groups.

	Dipper (n = 181)	Non-dipper (n = 121)	*P*
Age, yr	54.7 ± 12.6	55.2 ± 13.8	.717
Gender, male (%)	63 (34.8)	53 (43.8)	.115
Diabetes mellitus (%)	27 (14.9)	25 (20.7)	.195
Smoking (%)	71 (39.2)	53 (43.8)	.428
BMI, kg/m^2^	25.1 ± 3.2	25.7 ± 3.2	.146
Day SBP, mm Hg	143.0 ± 4.7	142.3 ± 4.5	.187
Day DBP, mm Hg	86.6 ± 5.2	87.3 ± 6.2	.303
Night SBP, mm Hg	122.5 ± 5.5	132.6 ± 6.4	**<.001**
Night DBP, mm Hg	75.1 ± 6.5	78.8 ± 8.7	**<.001**
24-h SBP, mm Hg	135.9 ± 3.6	136.6 ± 3.9	.103
24-h DBP, mm Hg	82.3 ± 5.7	83.3 ± 6.1	.134
LVEF (%)	60.6 ± 4.2	59.7 ± 4.8	.086
LVMI, g/m^2^	74.7 ± 23.8	80.6 ± 24.5	**.039**

Bold values indicate statistically significant results.

BMI = body mass index, DBP = diastolic blood pressure, LVEF = left ventricular ejection fraction, LVMI = left ventricular mass index, SBP = systolic blood pressure.

Laboratory variables of the study groups are presented in Table 2. No significant differences were found between 2 groups regarding laboratory variables (Table 2).

**Table 2:** Comparison of baseline laboratory parameters of the study groups.

	Dipper (n = 181)	Non-dipper (n = 121)	*P*
Glucose, mg/dL	98 (89–111)	100 (90–117)	.209
Urea, mg/dL	28 (22–35)	29 (24–36)	.194
Uric acid, mg/dL	4.8 (4.1–6.1)	5.2 (4.3–6.1)	.417
Creatinine, mg/dL	0.9 ± 0.2	0.9 ± 0.2	.483
Sodium, mEq/L	140.0 ± 2.3	139.9 ± 2.4	.676
Potassium, mEq/L	4.4 ± 0.4	4.4 ± 0.4	.613
Total cholesterol, mg/dL	224.7 ± 53.9	217.9 ± 48.5	.268
LDL-cholesterol, mg/dL	139 (112–166)	136 (106–160)	.305
HDL-cholesterol, mg/dL	55 (46–62)	53 (46–64)	.852
Triglyceride, mg/dL	126 (86–186)	124 (81–167)	.412
Leukocytes, ×10^3^/µL	7.6 ± 1.9	7.5 ± 2.0	.642
Hemoglobin, g/dL	13.5 ± 1.6	13.8 ± 1.6	.069
Platelets, ×10^3^/µL	255 (216–303)	262 (219–304)	.549
TSH, mU/L	1.8 (1.2–2.6)	1.7 (1.0–2.5)	.674

HDL = high density lipoprotein, LDL = low density lipoprotein, TSH = thyroid stimulating hormone.

### 3.2. Comparison of electrocardiographic (ECG) parameters and correlation analysis

On the other hand, comparison of the electrocardiographic variables is listed in Table 3. Non-dipper hypertensive patients had significantly higher QTc (417.6 ± 19.7 vs 413.1 ± 18.1, *P* = .042), TP-e (82.5 ± 17.4 vs 75.1 ± 14.7, *P* < .001) and frontal QRS-T angle (34 [16–64] vs 46 [19–92], *P* = .006) compared to the dipper hypertensive patients. These patients had also significantly higher ERS (1 [1–2] vs 2 [1–3], *P* < .001).

**Table 3:** Comparison of electrocardiographic variables of the study groups.

	Dipper (n = 181)	Non-dipper (n = 121)	*P*
Heart rate,/min.	77.1 ± 13.3	79.7 ± 10.9	.065
QRS transition zone ≥ V4 (%)	63 (34.8)	60 (49.6)	**.010**
QTc interval, ms.	413.1 ± 18.1	417.6 ± 19.7	**.042**
Tp-e interval, ms.	75.1 ± 14.7	82.5 ± 17.4	**<.001**
Frontal QRS-T angle	34 (16–64)	46 (19–92)	**.006**
ERS	1 (1–2)	2 (1–3)	**<.001**

Bold values indicate statistically significant results.

ERS = electrical risk score, QTc = corrected QT interval, Tp-e = T peak to T end interval.

A positive correlation was identified in the context of correlation analysis between ERS and nighttime SBP (*r* = 0.210, *P* < .001).

### 3.3. Independent predictors of non-dipper hypertension and ROC analysis

Independent predictors of non-dipper status, as identified through regression analyses, are presented in Table 4. Univariate and multivariate analyses were used to determine the independent predictors of non-dipper status. Left ventricular ejection fraction, LVMI, hemoglobin and ERS were detected as the possible predictor of non-dipper status in univariate analysis. However, ERS (OR: 1.935, 95% CI: 1.543–2.426, *P* < .001) was detected as the only independent predictor of non-dipper status in multivariate logistic regression analysis (Table 4).

**Table 4:** Independent predictors of non-dipper status according to the regression analyses.

	Univariate			Multivariate		
	**OR**	**95% CI**	***P***	**OR**	**95% CI**	***P***
Age	1.003	0.986–1.021	.716			
Gender, male	0.685	0.427–1.098	.116			
Diabetes mellitus	0.673	0.369–0.673	.197			
Smoking	0.828	0.519–0.828	.429			
BMI	1.055	0.981–1.135	.148			
LVEF	0.954	0.905–1.007	.088	0.965	0.908–1.025	.250
LVMI	1.010	1.000–1.020	.040	1.000	0.988–1.011	.946
Uric acid	1.058	0.912–1.228	.455			
Creatinine	1.590	0.436–5.790	.482			
LDL-cholesterol	0.997	0.991–1.002	.253			
WBC	0.972	0.861–1.096	.641			
Hemoglobin	1.144	0.989–1.323	.070	1.098	0.940–1.283	.238
Platelets	1.001	0.997–1.004	.705			
ERS	1.970	1.582–2.452	<.001	1.935	1.543–2.426	**<.001**

Bold value indicates statistically significant results.

BMI = body mass index, ERS = electrical risk score, LDL-cholesterol = low-density lipoprotein cholesterol, LVEF = left ventricular ejection fraction, LVMI = left ventricular mass index, WBC = white blood cell count.

**Figure 1.**
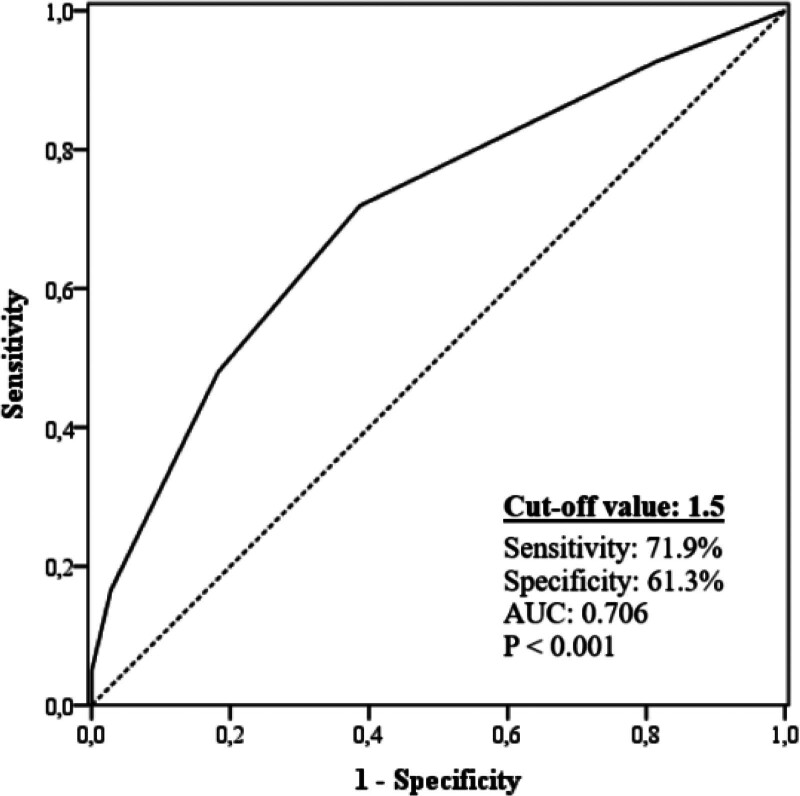
ROC curve of electrical risk score for predicting non-dipper status. ROC = receiver operating characteristic.

**Figure 2.**
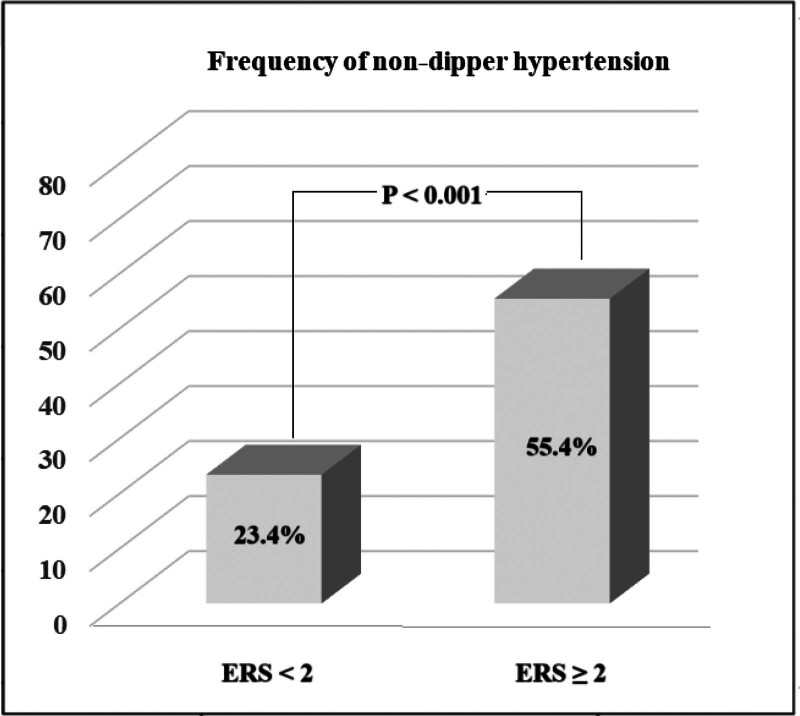
The frequency of non-dipper status in patients with ERS < 2 and ≥ 2. ERS = electrical risk score.

**Figure 3.**
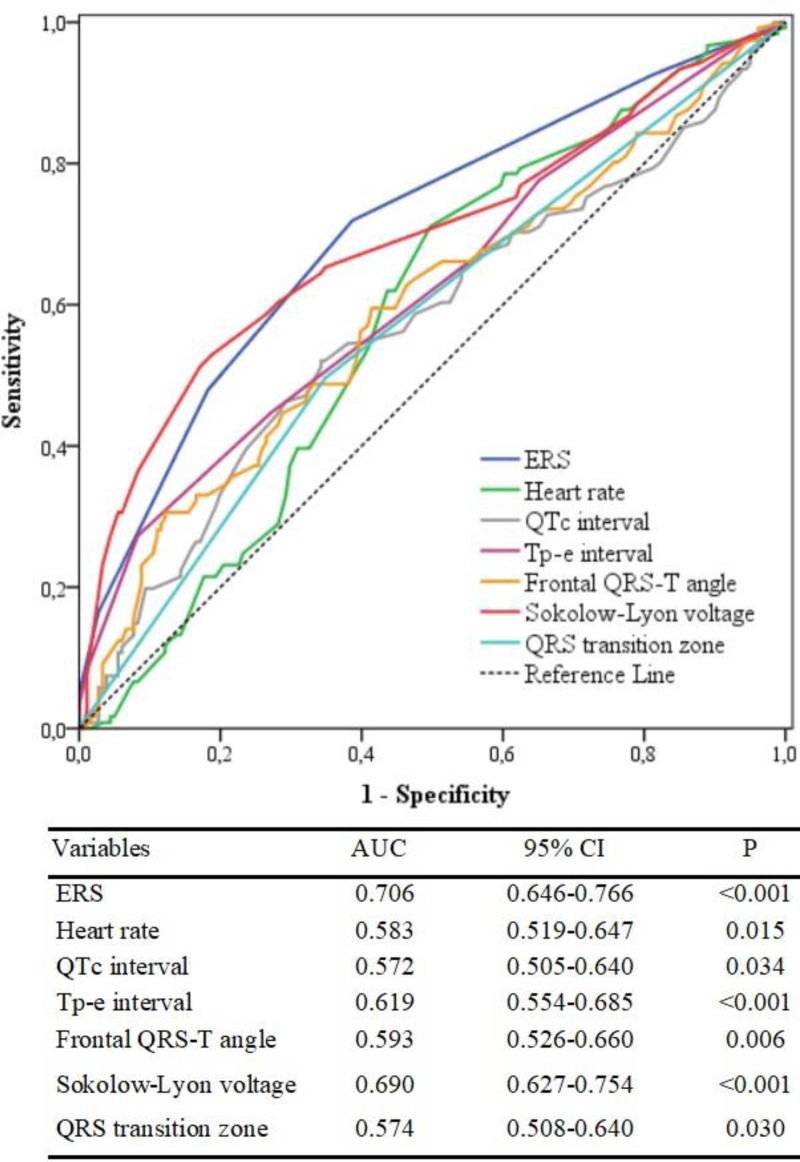
ROC curve and AUC value of each ECG parameter for predicting the non-dipper hypertension. AUC = area under the curve, ECG = electrocardiography, ROC = receiver operating characteristic.

ROC analysis was performed to obtain the cutoff value and AUC of the ERS for predicting non-dipper hypertension. ERS ≥ 1.5 predicted the non-dipper status with a sensitivity of 71.9% and specificity of 61.3% (AUC: 0.706, *P* < .001) (Fig. 1). When our study population was divided into 2 groups according to this cutoff value (those patients with ERS < 2 and ERS ≥ 2), it was detected that the frequency of non-dipper status was significantly higher in patients with ERS ≥ 2 than in patients with ERS < 2 (Fig. 2). In addition, the AUC of each ECG parameter and ERS for predicting non-dipper status are shown in Figure 3. We found that ERS had the highest AUC value among all ECG parameters for predicting the non-dipper HT (Fig. 3).

## 4. Discussion

The aim of this study was to investigate the relationship between the ERS and non-dipper hypertension in patients with hypertension. The main result of our study was that ERS was an independent predictor of non-dipper status in patients with hypertension. In addition, as ERS increased, night SBP increased significantly. To our knowledge, this is the first study regarding the independent relationship between ERS and non-dipper hypertension.

Cardiovascular system is one of the main targets of elevated blood pressure (BP), and ventricular myocardium is the most susceptible cardiac structure to hypertension mediated end-organ damage.^[1]^ If hypertension is not effectively managed, it can lead to the development of end-organ damage and the onset of adverse cardiovascular diseases. BP follows a 24-hour circadian rhythm, with a normal decline of more than 10% during the night. If this decrease in BP is <10%, this is called a non-dipper pattern.^[1,3]^ Previous studies showed that non-dipper hypertension was associated with increased end-organ damage and higher risks of cardiovascular and cerebrovascular diseases.^[21–23]^ In addition, it was found that non-dipper hypertension patients had a higher frequency of left ventricular hypertrophy, conduction disturbances and myocardial repolarization impairment.^[24–26]^ It is hypothesized that a BP profile which fails to demonstrate a sufficient physiological decrease during the nocturnal period may result in structural alterations, particularly within the left ventricle, via the renin-angiotensin-aldosterone system.^[27–29]^ In advanced stages, the remodeling process gives rise to diastolic dysfunction and end-organ damage, resulting in increased LVMI and consequently a heightened risk of cardiovascular events, hospitalizations and mortality.^[30–32]^ Similar to these results, we also found that LVMI was significantly higher in non-dipper pattern compared to the dipper pattern.

Previous studies have sought to evaluate the risk in patients with non-dipper hypertension through the utilization of specific ECG parameters obtained during clinical visits.^[6,33–35]^ Nevertheless, the majority of studies that have focused on ECG in non-dipper hypertension have concentrated on a single ECG parameter, investigating its impact on clinical outcomes. To the best of our knowledge, no study has yet explored the ERS in non-dipper hypertension patients or assessed its influence on clinical outcomes. The value of our study lies in its comprehensive evaluation of all ECG parameters contributing to ERS and their subsequent effects on clinical outcomes.

Tp-e, QTc and frontal QRS-T angle are the parameters of myocardial depolarization and repolarization. Previous studies showed that these parameters alone were significantly increased in patients with non-dipper hypertension.^[35–38]^ We also found that non-dipper hypertensive patients had significantly higher Tp-e, QTc and frontal QRS-T angle compared to the dipper hypertensive patients. These results suggest that patients with non-dipper hypertension had higher heterogeneity in cardiac electrical activity and myocardial stress. This situation leads to myocardial structural changes and fibrotic changes due to increased left ventricular pressure, and cause delays in ventricular repolarization, inflammation, vascular dysfunction, autonomic imbalance and electrical axis deviations.^[39]^

The QRS transition zone is a readily discernible electrocardiographic parameter, readily identified on a standard ECG. It is an indicator that is employed for the purpose of assessing the electrical activity of the heart, with a particular focus on the ventricular conduction system. The QRS transition zone is defined as the presence of a delayed QRS transition zone in leads V4 or higher. A previous study demonstrated that a delayed QRS transition zone is associated with an increased risk of cardiovascular disease.^[19]^ The QRS transition zone has yet to be studied in non-dipper hypertensive patients; however, delayed QRS transition zones have been demonstrated to be markedly elevated in this patient group.^[19]^ In our study, we found that the frequency of delayed QRS transition zone was significantly higher in patients with non-dipper hypertension. This indicates that sustained elevated BP during the nocturnal period, augmented ventricular pressure resulting from hypertrophy or fibrosis, may precipitate delayed ventricular conduction and contribute to electrical abnormalities within the cardiac muscle.

The 12-lead ECG plays an important role for risk stratification in patients with hypertension. Although numerous studies have been conducted on the correlation between ECG parameters and hypertension, ECG parameters have been studied separately in most of these studies, and there is no study in which all these ECG parameters have been studied together. ERS is a novel risk scoring system and introduced by Aro et al.^[13]^ It consists of 6 different ECG parameters and shows the imbalanced neuro-autonomic control (heart rate, QTc, and Tp-e), the repolarization alterations (QTc, QRS-angle, and Tp-e), and cardiac hypertrophy (QTc, QRS-angle, QRS transition, and Tp-e). Therefore, it can provide very detailed information to the clinician. To date, no study has investigated the ERS in hypertension. We found that ERS was an independent predictor of non-dipper pattern in hypertensive patients. Also, it was detected that ERS was positively correlated with nighttime SBP. These results suggest that ERS is an important and simple electrocardiographic tool for detecting the non-dipper status in patients with hypertension.

### 4.1. Limitations

The main limitations of our study include the relatively small sample size and its retrospective single-center design, which may limit the generalizability of our findings. We did not perform follow-up for morbidity, arrhythmic events, or other cardiovascular outcomes, which would have provided valuable prognostic insights regarding ERS. In addition, we did not adjust for potential confounding variables such as the type and timing of antihypertensive medication, which could have influenced both BP patterns and ECG parameters.

Furthermore, inter-rater reliability of the ERS measurements was not assessed, which may affect the reproducibility of the ECG scoring. We also did not perform internal validation (e.g., bootstrapping or cross-validation) for the ERS cutoff point, and our findings have yet to be externally validated in independent cohorts.

Prospective, multicenter studies with larger patient populations and systematic follow-up are needed to confirm the clinical utility and generalizability of ERS in predicting non-dipper hypertension.

## 5. Conclusion

The ERS is an easily obtainable parameter from the surface of ECG. Our study reported an important relationship between ERS and non-dipper hypertension. We found that ERS was an independent predictor of non-dipper pattern in patients with hypertension. Therefore, we suggest that ERS may be used as a simple and beneficial tool for detecting the non-dipper pattern in patients with hypertension.

## Author contributions

**Conceptualization:** Ali Nizami Elmas, Halil Fedai, Zulkif Tanriverdi.

**Data curation:** Ali Nizami Elmas, Zulkif Tanriverdi.

**Formal analysis:** Ali Nizami Elmas, Halil Fedai.

**Investigation:** Ali Nizami Elmas, Halil Fedai, Zulkif Tanriverdi.

**Methodology:** Ali Nizami Elmas, Halil Fedai, Zulkif Tanriverdi.

**Project administration:** Ali Nizami Elmas.

**Resources:** Ali Nizami Elmas.

**Software:** Ali Nizami Elmas.

**Supervision:** Zulkif Tanriverdi.

**Validation:** Ali Nizami Elmas, Zulkif Tanriverdi.

**Visualization:** Ali Nizami Elmas, Halil Fedai, Zulkif Tanriverdi.

**Writing – original draft:** Ali Nizami Elmas, Zulkif Tanriverdi.

**Writing – review & editing:** Ali Nizami Elmas, Zulkif Tanriverdi.
